# Comparative metabolomics in vanilla pod and vanilla bean revealing the biosynthesis of vanillin during the curing process of vanilla

**DOI:** 10.1186/s13568-017-0413-2

**Published:** 2017-06-05

**Authors:** Fenglin Gu, Yonggan Chen, Yinghua Hong, Yiming Fang, Lehe Tan

**Affiliations:** 1 Spice and Beverage Research Institute, CATAS, Wanning, 571533 Hainan China; 2 College of Tropical Biology and Agronomy, Hainan Tropical Ocean University, Sanya, 572022 Hainan China; 3 Key Laboratory of Genetic Resources Utilization of Spice and Beverage Crops, Ministry of Agriculture, Wanning, 571533 Hainan China

**Keywords:** Vanilla curing, LC–MS, Metabolomics, Vanillin biosynthesis

## Abstract

High-performance liquid chromatography–mass spectrometry (LC–MS) was used for comprehensive metabolomic fingerprinting of vanilla fruits prepared from the curing process. In this study, the metabolic changes of vanilla pods and vanilla beans were characterized using MS-based metabolomics to elucidate the biosynthesis of vanillin. The vanilla pods were significantly different from vanilla beans. Seven pathways of vanillin biosynthesis were constructed, namely, glucovanillin, glucose, cresol, capsaicin, vanillyl alcohol, tyrosine, and phenylalanine pathways. Investigations demonstrated that glucose, cresol, capsaicin, and vanillyl alcohol pathway were detected in a wide range of distribution in microbial metabolism. Thus, microorganisms might have participated in vanillin biosynthesis during vanilla curing. Furthermore, the ion strength of glucovanillin was stable, which indicated that glucovanillin only participated in the vanillin biosynthesis during the curing of vanilla.

**Electronic supplementary material:**

The online version of this article (doi:10.1186/s13568-017-0413-2) contains supplementary material, which is available to authorized users.

## Introduction

Vanilla is a tropical orchid, which originated in Mexico (Lubinsky et al. 2008). It is one of the most important and popular aromatic compound used in food, beverages, and cosmetics (Kaur and Chakraborty 2013; Korthou and Verpoorte 2007). The fruit of a fully grown and mature vanilla is called vanilla pod. Vanilla pods are flavorless, but develop a characteristic aroma during the curing process. Traditional curing process generally comprises four steps, namely killing, sweating, drying, and conditioning. Vanilla bean refers to the vanilla pod after the curing process (Frenkel et al. 2011; Mariezcurrena et al. 2008; Sreedhar et al. 2009).

Natural vanilla flavor comprises a large number of aromatic compounds, including the principal flavor component vanillin and over 200 other volatile compounds with delicate sweet fragrances (Sharp et al. 2012). Vanillin content varies with different curing processes used around the world. Madagascar produces the best quality vanilla, with a vanillin content of 2.0–3.4%, whereas the Indian vanilla contains 1.0–2.0% vanillin (Ranadive 1994; Westcott et al. 1994; Röling et al. 2001). Despite the fact that vanillin is the most popular flavor and probably the most prevalent natural plant product, it is also an extremely simple molecule. Since the vanillin biosynthesis pathway remains unclear, various attempts to modernize and improve the curing process have been conducted, involving oven drying, solar drying, and enzyme treatment (Dignum et al. 2001). However, the biosynthesis of vanillin is not efficient, nor could the quality of the vanilla bean be easy to control.

Metabolomics, a powerful approach in investigating the characteristics of the low- molecular-weight metabolite present in a biological sample, has become an important part of systems biology, complementing genomics and proteomics (Sasaki et al. 2016). The main analytical techniques applied in metabolomics analysis are nuclear magnetic resonance (NMR) spectroscopy, gas chromatography–mass spectrometry (GC–MS), and liquid chromatography–mass spectrometry (LC–MS). These techniques facilitate analysis of a wide range of metabolites with diverse physicochemical properties occurring at different concentration levels (Dunn and Ellis 2005). However, GC–MS may be prone to confounding factors introduced by the sample derivatization process or thermal degradation of metabolites at elevated temperatures (Xu et al. 2010). Furthermore, NMR may be biased toward the detection of large abundance metabolites and is the least sensitive of the three techniques. LC–MS provides high sensitivity and be capable to detect a wide range of metabolites (Chen et al. 2011). Nevertheless, it is susceptible to retention time (RT) drift and matrix effects related to electrospray ionization (ESI). The downstream data processing is time-consuming and complex. Advanced data processing software algorithms and multivariate chemometric tools are needed to process and interpret data obtained in LC–MS-based metabolomics.

A previous study has shown that the colonizing *Bacillus* isolates produce β-d-glucosidase, which mediates glucovanillin hydrolysis and influences flavor formation (Chen et al. 2015). The study further indicated that the curing process would result in metabolite variation. However, only few reports on the effects of curing on the metabolite variations of vanilla have been conducted (Frenkel et al. 2011; Gallage et al. 2014; Dignum et al. 2002; Palama et al. 2010, 2012). In this study, we used liquid chromatography-mass spectrometry (LC–MS) and multivariate chemometric methods to characterize the metabolic changes between vanilla pod and vanilla bean, identifying characteristic metabolites, and interpreted these changes in order to reveal the biosynthesis pathway of vanillin.

## Materials and methods

### Plant materials and chemicals

Vanilla pods were collected in Hainan, China, and cured by hot air processing (Dong et al. 2014). HPLC grade methanol was purchased from Fisher Scientific (Fair Lawn, NJ, USA), and HPLC-grade acetonitrile and reagent grade formic acid were obtained from Merck KGaA (Darmstadt, Germany). Distilled water was purified with Milli-Q Integral System Millipore (Bedford, MA, USA).

### Sample preparation

Thirty vanilla pods and cured vanilla beans were collected for LC–MS analysis. The sample was crushed and placed in a 96-well plate, then precipitated using acetonitrile/methanol (50:50, v/v) solvent. After vacuum pump, each well was washed with 180 μL of the precipitate rinsing solvent. After drying the sample, 200 μL mixtures were used for extracting small molecular compounds, and the mixture was composed of 5% acetonitrile solution/methanol (70:30, v/v). Finally, the mixture was used for LC–MS analysis.

### Non-targeted metabolic profiling spectral acquisition

The LC–MS system was run in gradient mode. Solvent A was 5% (v/v) formic acid/mixture (10% methanol/90% water), and solvent B was acetonitrile. The flow rate was 0.2 mL/min. The total injection volume for analysis was 10 μL. A C-18 column (150 mm × 2.1 mm, 5 μm, Waters, USA) was used for all analyses. The gradient was as follows: 0–2 min, 5% B; 2–3 min, 7% B; 3–4 min, 8% B; 4–5 min, 70% B; 5–9 min, 90% B; 9–11 min, 95% B; 11–14 min, 95% – 5% B; and 14–15 min, 5% B.

LC–MS data was acquired using high performance-liquid chromatography (HPLC) system (LC-20AD, Shimadzu Co., Kyoto, Japan) coupled online to a LTQ Orbitrap Velos instrument (Thermo LTQ-Orbitrap Velos, Bremen, Germany) set at 30,000 resolution. Sample analysis was carried out in positive ion modes. The mass scanning range was 50–1000 m/z, the total retention time was 1200 s, and the capillary temperature was 350 °C. Nitrogen sheath gas was set at a flow rate of 30 L/min. Nitrogen auxiliary gas was set at a flow rate of 10 L/min and the spray voltage was set to 4.5 kV.

### Data analysis

For the extraction of LC–MS raw data, Xcalibur native acquisition was performed with XCMS. All data were processed by local XCMS with the following parameters: signal/noise threshold = 3, ppm = 10, peak width = (20, 50), and prefilter = (3, 1000). Retention time correction was performed with the standard obiwarp algorithm in XCMS with prfostep = 1. To correct the MS response shift during the run, the raw data were normalized against total integration values. Principal component analysis (PCA), orthogonal partial least squares discriminant analysis (OPLS-DA), and hierarchical cluster analysis (HCA) were performed by R-2.14.2. The VIP value and the significance were expressed by using Student’s t test of R-2.14.2. VIP > 1 and *p* < 0.05 were considered significant. Pathway Builder Tool 2.0 was used to build the vanillin metabolic pathways.

### Metabolite identification

KEGG.gff, HMDB.gff, HMDB-SERUM.gff, LIPID.gff, and MetaCyc.gff metabolism database were used to identify metabolites in the LC–MS chromatograms. For LC–MS data, the putative identities of each ion were first given within XCMS by matching features in the databases with the following parameters: ppm = 20, adducts = [M+H]^+^, [M+NH_4_]^+^, [M+Na]^+^, and [M+K]^+^ in the positive ion mode. Furthermore, the potential molecule formulas of each ion were extracted from a database based on accurate mass. The metabolites were identified based on accurate mass, fragmentation pattern, and retention time.

## Results

### Metabolic profiles of vanilla

**Fig. 1:**
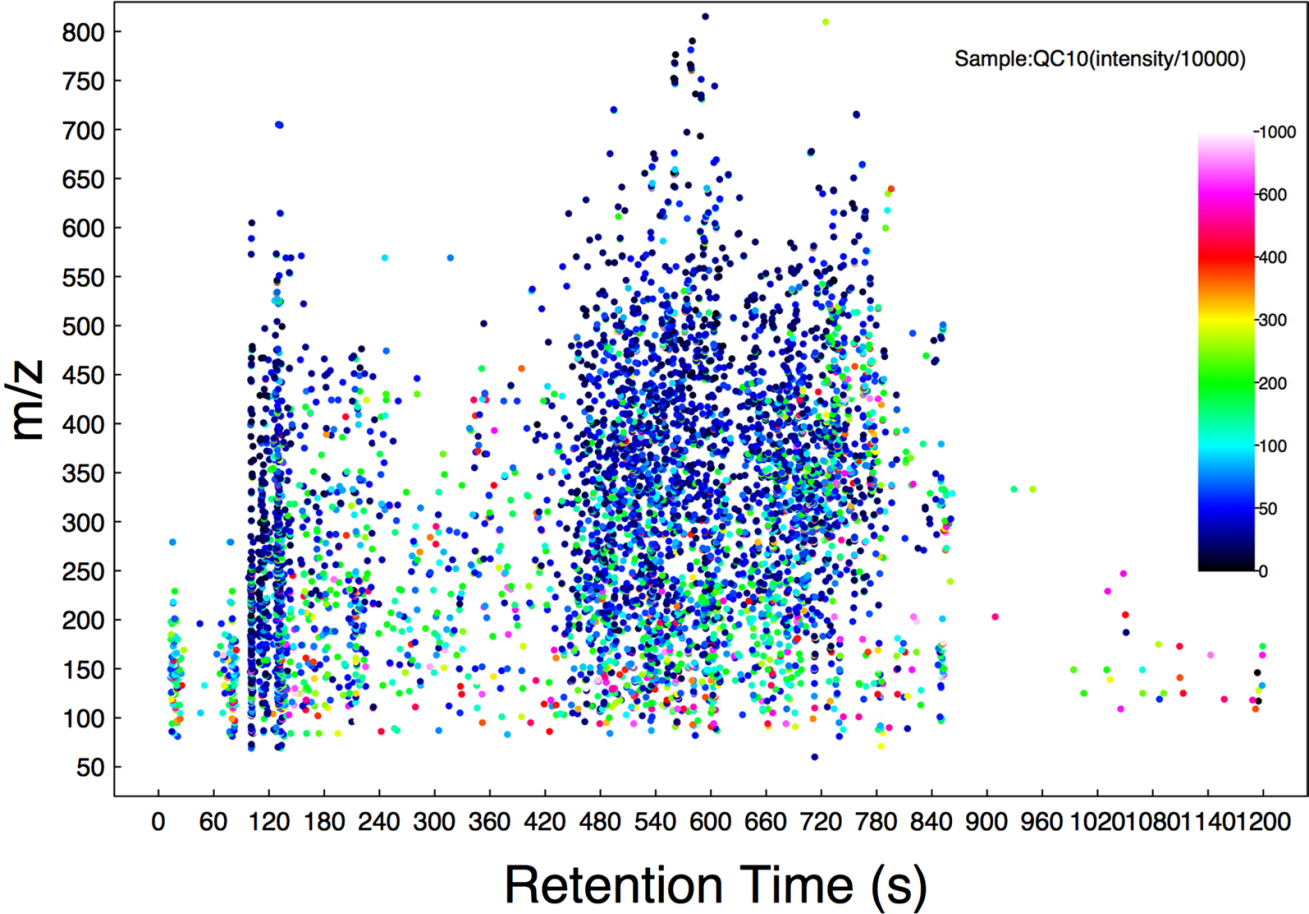
A chromatogram of total ions

The extracts of vanilla samples were analyzed via the LC–MS technique, allowing the analyses of a wide range of untargeted compounds. The representative LC–MS total ion current (TIC) chromatographs of the extracts obtained from vanilla pods and vanilla beans are shown in Fig. 1. The abscissa and ordinate represent the retention time and m/z value, respectively. Each point in the graph represents an ion. The color of each point corresponds to the ion signal intensity, following black > blue > green > red > white, with the ion signal intensity gradually increasing. The main m/z values were in the range of 50–800, and the retention time was concentrated at 60–900 s.

### Metabolic variations between vanilla pod and vanilla bean

**Fig. 2:**
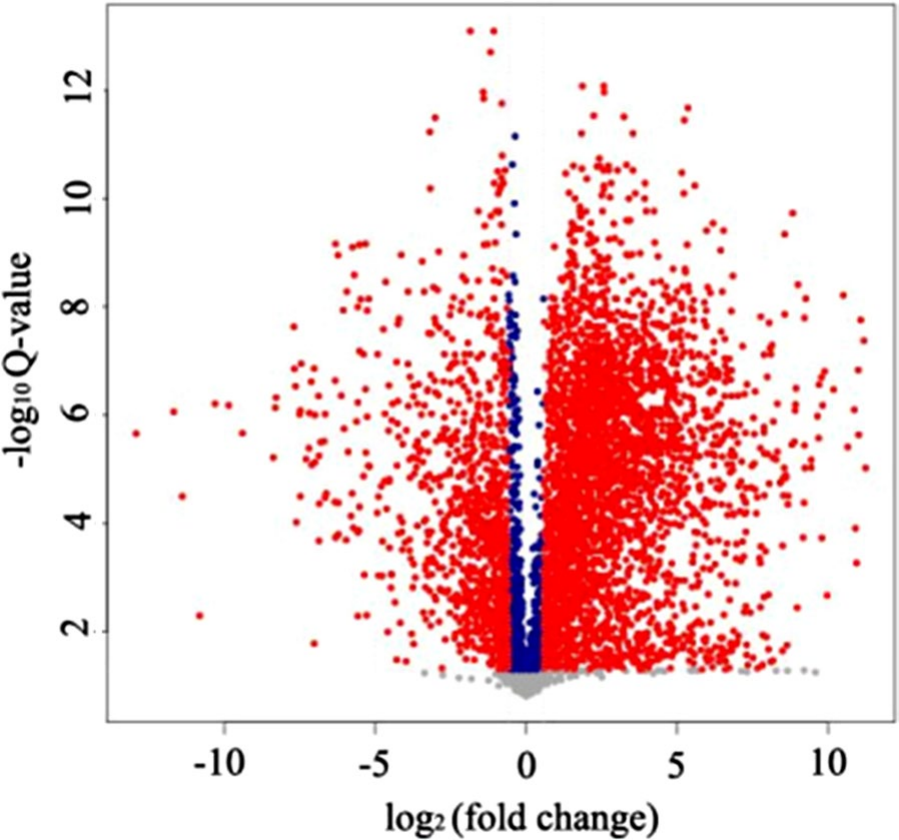
Volcano plot between vanilla pods and vanilla beans [variables in *red* are significant (Q < 0.05), and variables in *blue* are not significant]

For each m/z value, there is a LOESS value in the comparison group, and the average LOESS value of each sample was obtained. The ratio of LOESS value was taken for each m/z corresponding to the change fold (differential multiple), and the change fold greater than 1.5 or less than 0.67 of the m/z were selected. The logarithm of these change folds was used as the horizontal coordinate, and Q value as the vertical coordinate mapping. Red dots represent significantly different m/z, for 1723, accounting for 30.51%; the blue points are significantly similar m/z, for 635, accounting for 11.24%; and gray points represent non-significant m/z, for 3290, accounting for 58.25% (Fig. 2).

**Fig. 3:**
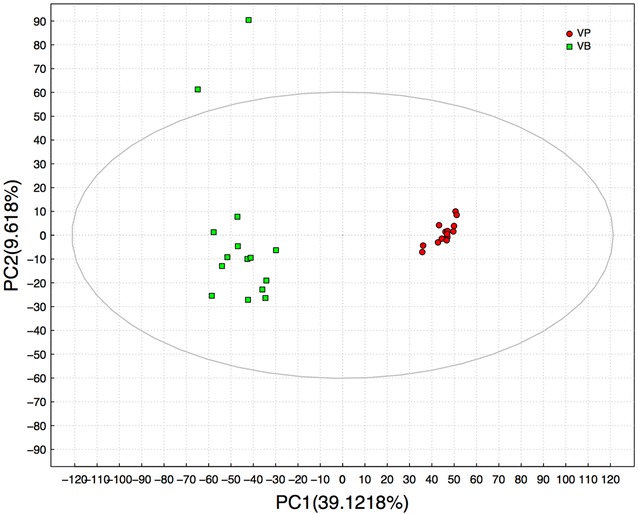
PCA score separating vanilla pods and vanilla beans away from each other (*VP* vanilla pods, *VB* vanilla beans)

PCA can extract chemical information objectively, which has been well established for discrimination and grouping in analyzing metabolic profiles. PCA developed a visual plot for the evaluation of similarities and differences between the metabolic profiles of vanilla pods and vanilla beans on the basis of metabolites. Based on a correction of 0–30% RSD (QC sample not included), the LOESS value was used for PCA analysis (Fig. 3). Principal component 1 (PC1) accounted for the major differences in variances, whereas the principal component 2 (PC2) accounted for the minor differences. Fifteen vanilla pods were clustered together, which indicated that the metabolites were generally similar. On the contrary, vanilla beans were scattered, implying that the metabolites were quite different. Furthermore, vanilla beans showed significant differences from vanilla pods. The results showed that vanilla beans were greatly distinct even though they were cured from the same vanilla pods.

**Fig. 4:**
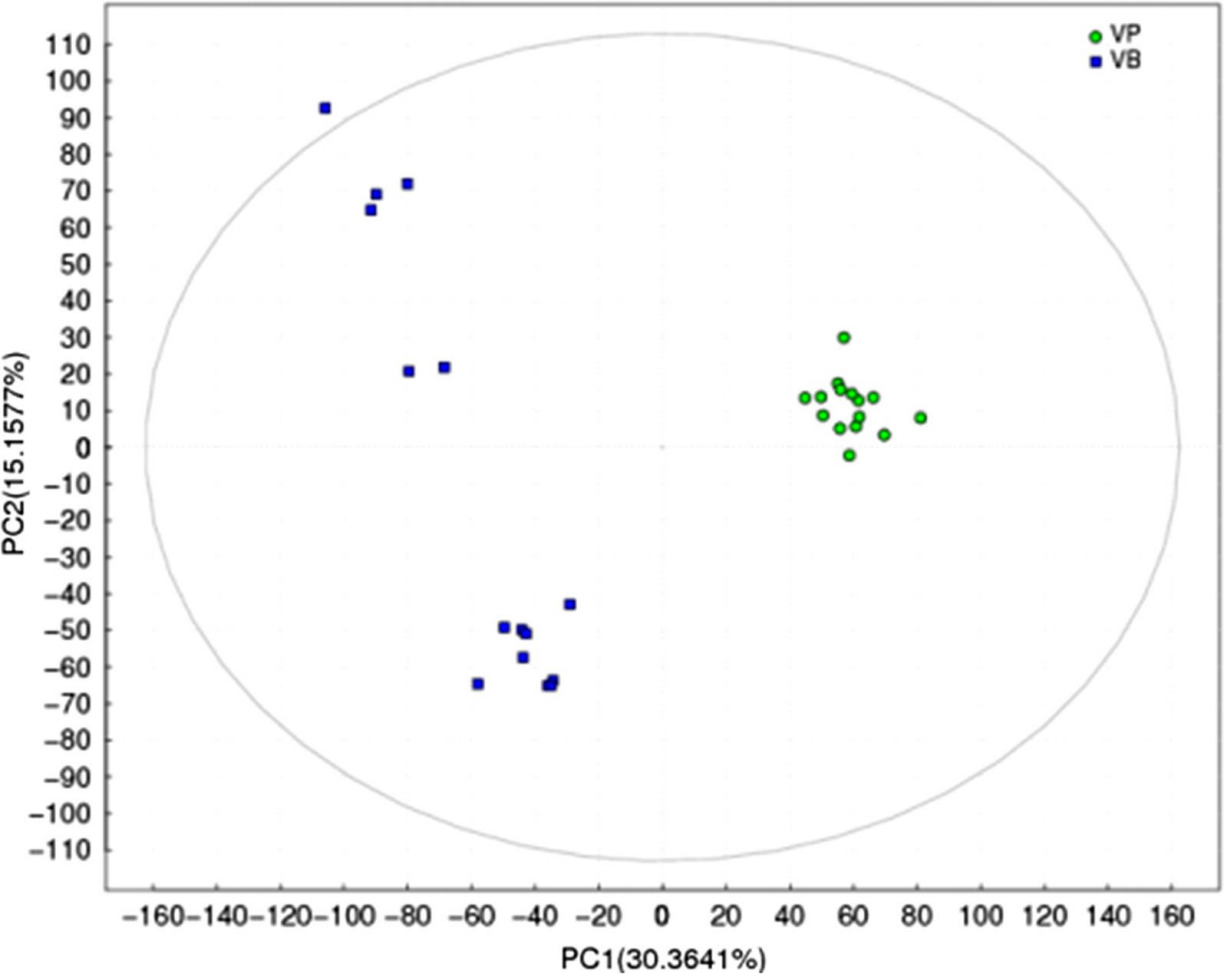
PLS-DA score separating vanilla pods and vanilla beans away from each other (*VP* vanilla pods, *VB* vanilla beans)

The metabolites exceeding in the PLS-DA (partial least squares-discriminant analysis) model and with a correction of 0–30% RSD data were selected to focus on the important marker metabolites distinguishing between vanilla pods and vanilla beans. The m/z group corresponding to the LOESS value was compared using OSC (orthogonal signal correction) method to remove part of the noise, and then carried out by PLS-DA analysis. The results of the PLS-DA analyses were similar as PCA analysis. Vanilla beans considerably differed from each other, and they were also different from vanilla pods (Fig. 4).

**Fig. 5:**
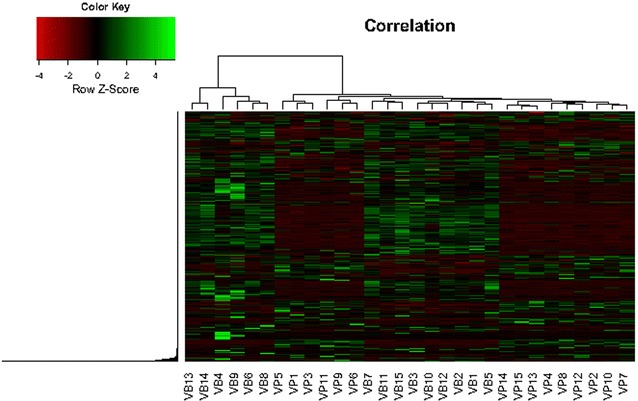
Heat map of cluster for vanilla pods and vanilla beans

A heat map was constructed based on the detection of all samples, with a total of 5648 m/z values (Fig. 5). The results showed that the same group samples were quite similar, indicating the significant differences of metabolites in the fermentation of vanilla pods and vanilla beans.

### The speculated biosynthesis pathway of vanillin

According to the results of the comparison of the m/z values with the database, 6822 kinds of compounds were identified where 21 compounds related to vanillin synthesis were identified based on the reported biosynthesis pathway of vanillin. The ion strength of individual components changed and was related to vanillin biosynthesis as shown in Additional file 1: Figure S1. The ion strength of glucovanillin was stable in vanilla pods and vanilla beans, whereas the ion strength of most other compounds exhibited divergence.

**Fig. 6:**
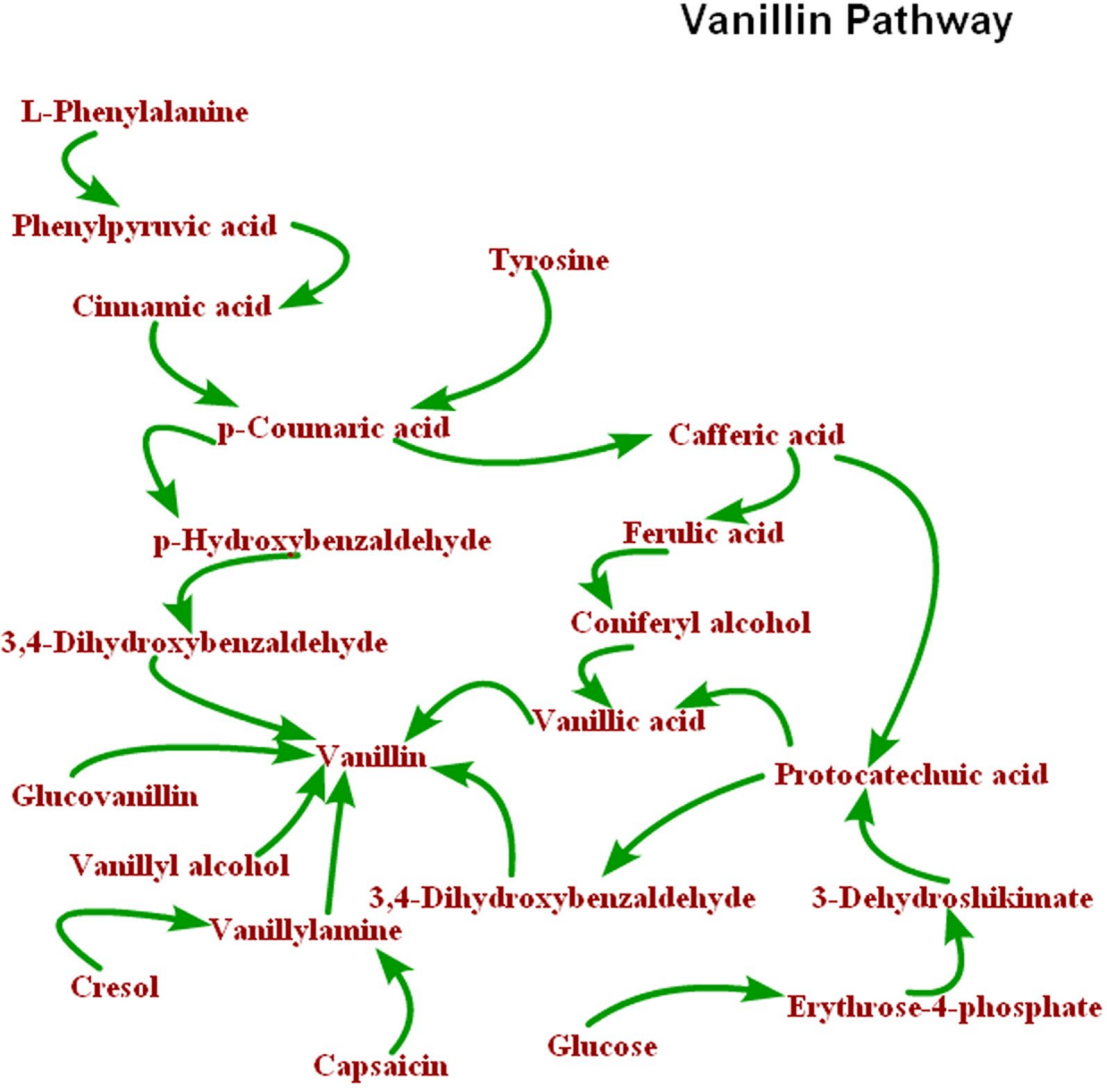
Some presumed metabolic pathways of vanillin biosynthesis (cited from: the pathway of glucose, capsaicin, cresol, vanillyl alcohol, l-phenylalanine, tyrosine cited from Kaur and Chakraborty 2013; and the pathway of glucovanillin cited from Dignum et al. 2004)

The biosynthesis pathway of vanillin during fermentation was constructed based on the precursors of vanilla synthesis, as shown in Fig. 6. Previous studies reported that vanillin originated from β-d-glucosidase hydrolysis of glucovanillin (Dignum et al. 2004) or ferulic acid decarboxylation (Gallage et al. 2014). In our study, these two mentioned pathways were also found. In addition, the precursors of ferulic acid were also detected, namely l-phenylalanine and tyrosine. Moreover, this study also found four more vanillin synthesis pathways, which, in our knowledge, were not reported to occur in vanilla curing. These pathways distributed in a wide range of microbial metabolism (Kaur and Chakraborty 2013). The four newly-found pathways of vanillin biosynthesis were as follows: shikimic acid pathway of glucose by de novo synthesis; and oxidation of cresol, capsaicin, and vanilla alcohol.

## Discussion

The curing process is an essential procedure for the production of vanilla flavor. This process induces destruction of tissue organization and reduces vanilla pod moisture. Furthermore, it creates conditions which allow the free flow of compartmentalized cellular constituents, resulting in enzyme-substrate interactions (Frenkel et al. 2011). Various biochemical and chemical reactions, such as esterification, etherification, and oxidative degradation, take place during this period to produce volatile compounds (Uzio and Derbesy 1989). However, the variations, especially those relating to the metabolism of vanilla fruits, still remain poorly constrained (Palama et al. 2009, 2011). Previous studies mainly focused on volatile compounds of vanilla beans but not on the overall metabolic fingerprints of vanilla fruits (Zhang and Christoph 2012; Pérez-Silva et al. 2006). This study applied untargeted metabolomic and chemometric analysis, indicating a notable differentiation between vanilla pods and vanilla beans. As shown in Figs. 3 and 4, PCA and PLS-DA demonstrated a clear and statistically significant separation of vanilla pods and vanilla beans. The substantial changes were observed in the metabolite composition of vanilla beans. Results showed that the curing process could give rise to metabolite changes in vanilla beans even though they contained similar metabolites in vanilla pods. This could be attributed to the effect of the different environmental microbes during the curing process. As shown in Additional file 1: Figure S1, the stability of glucovanillin ion strength showed that it was mainly involved in the metabolic pathway of vanillin biosynthesis and did not participate in other metabolic ways during the curing process, and the ion strength divergence of the other compounds indicated they not only participated in the vanillin biosynthesis, but were also involved in other flavors formation by the microbial metabolism.

Biosynthesis of vanillin is of interest to researchers for two main reasons: the relationship of vanillin with the mechanisms of the formation of benzoic acids and with the phenylpropanoid pathway; and the commercial importance of vanillin and the possibilities of producing the compound by biotechnological routes (Kaur and Chakraborty 2013). Vanillin in green pods is present exclusively in conjugated form, principally as the glucovanillin, and the beans display no trace of the characteristic vanilla flavor at this stage (Odoux 2006; Walton et al. 2003). Vanillin only develops during the curing process. One of the most obvious aspects of curing is that glucovanillin reacts with β-d-glucosidase to form vanillin (Kanisawa et al. 1994; Ramachandra and Ravishankar 2000; Dignum et al. 2001). In addition, Zenk (1965) reported the results of radioactive label ferulic and vanillic acids, and proposed a route by which vanillin was derived from ferulic acid. A quite different route was later proposed based on enzyme assay (Reference needed, I guess it’s Dixon 2011?). According to this route, vanillin was formed from coumaric acid by non-oxidative chain shortening or via any one of three Coenzyme A esters by β-oxidation. However, to our knowledge, this is the first time that the glucose, cresol, capsaicin, and vanillyl alcohol pathways were proposed, which implied that vanillin biosynthesis was not as simple as it seemed. Furthermore, the pathways and mechanisms elucidated in this study could aid future studies on the production of vanillin.

Various metabolic pathways of vanillin biosynthesis were found in bacteria, fungi, plant cells, and genetically engineered microorganisms. These metabolisms included the production of vanillin from lignin, eugenol, isoeugenol, phenolic stilbenes, ferulic acid, and amino acids (Zhao et al. 2005). However, only *p*-coumaric acid and ferulic acid were found as precursors in the vanilla and in vanilla cell cultures, which were involved in the shikimate and phenylalanine pathways (Gallage et al. 2014). In this study, three other biosynthesis pathways were evidenced: glucose, cresol and capsaicin oxidation. A flavoprotein vanillyl alcohol oxidase (vaoA) was essential for creosol and capsaicin to produce vanillin (Kaur and Chakraborty 2013). VaoA was found in a wide range of microorganisms, such as *Dictyostelium discoideum*, *Cavia porcellus*, *Saccharomyces cerevisiae*, *Candida albicans*, and *Streptococcus pneumoniae* (Leferink et al. 2008). Similarly, 3-dehydroshikimate dehydratase was essential in the glucose pathway via de novo biosynthesis, which was found in *Emericella nidulans*, *Neurospora crassa*, *Klebsiella pneumoniae*, *Vigna mungo*, *Bacillus thuringiensis*, and *Podospora pauciseta* (Lamb et al. 1992; Draths and Frost 1995; Fox et al. 2008; Hansen et al. 2009). However, only few reports were known regarding the presence of two enzymes in Orchidaceae (Hansen et al. 2009).

The metabolic profiles of vanilla pods and vanilla beans illustrated that the metabolome of vanilla fruits were mainly dominated by 5648 m/z. According to the m/z analysis, the vanilla pods showed significant differences from vanilla beans. In addition, 21 precursors related to vanillin synthesis were identified, and 7 pathways of vanillin biosynthesis were constructed, including glucovanillin, glucose, cresol, capsaicin, vanillyl alcohol, tyrosine, and phenylalanine pathways. To the best of our knowledge, the glucose, cresol, capsaicin, and vanillyl alcohol pathways have not yet been reported in any vanilla curing process. However, these pathways have wide range of distribution in microbial metabolism. Therefore, microorganisms may have participated in vanillin biosynthesis during the curing of vanilla.

## Additional file

**Additional file 1: Figure S1.** A part of components change in the process and relate to the vanillin biosynthesis. a. Erythrose-4-phosphate; b. Glucose; c. p-Hydroxybenzaldehyde; d. 3,4-Dihydroxybenzaldehyde; e. Protocatechuic acid; f. Cresol; g. 3-Dehydroshikimate; h. Vanillin; i. Vanillic acid; j. Vanillyl alcohol; k. Vanillylamine; l. Cinnamic acid; m. Phenylpyruvic acid, p-Coumaric acid; n. Cafferic acid; o. L-Phenylalanine; p. Tyrosine; q. Ferulic acid; r. Coniferyl alcohol; s. Glucovanillin; t. Capsaicin.

## Abbreviations

LC–MS: high-performance liquid chromatography–mass spectrometry
NMR: nuclear magnetic resonance
GC–MS: gas chromatography–mass spectrometry
RT: retention time
ESI: electrospray ionization
HPLC: high performance-liquid chromatography
PCA: principal component analysis
OPLS-DA: orthogonal partial least squares discriminant analysis
HCA: hierarchical cluster analysis
TIC: total ion current
PLS-DA: partial least squares-discriminant analysis
OSC: orthogonal signal correction
vaoA: flavoprotein vanillyl alcohol oxidase
