# Supplementary material for: Comparative metabolomics in vanilla pod and vanilla bean revealing the biosynthesis of vanillin during the curing process of vanilla
Source: AMB Express. 2017 Jun 5;7:116. doi: 10.1186/s13568-017-0413-2 (PMC5459784; doi:10.1186/s13568-017-0413-2)
Supplement: Supplementary file 1 — Additional file 1: Figure S1. A part of components change in the process and relate to the vanillin biosynthesis. a. Erythrose-4-phosphate; b. Glucose; c. p-Hydroxybenzaldehyde; d. 3,4-Dihydroxybenzaldehyde; e. Protocatechuic acid; f. Cresol; g. 3-Dehydroshikimate; h. Vanillin; i. Vanillic acid; j. Vanillyl alcohol; k. Vanillylamine; l. Cinnamic acid; m. Phenylpyruvic acid, p-Coumaric acid; n. Cafferic acid; o. L-Phenylalanine; p. Tyrosine; q. Ferulic acid; r. Coniferyl alcohol; s. Glucovanillin; t. Capsaicin. [file 13568_2017_413_MOESM1_ESM.docx]

d

c

b

a


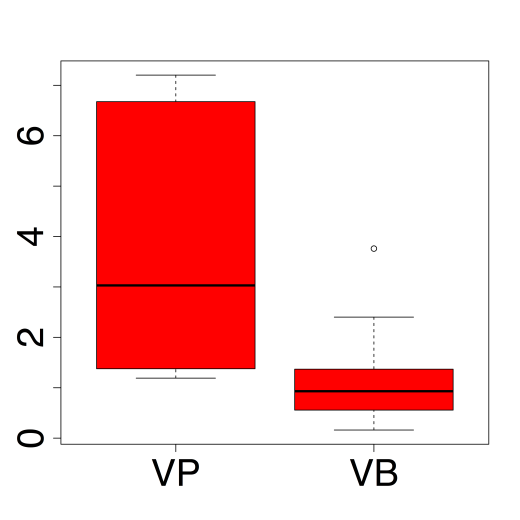

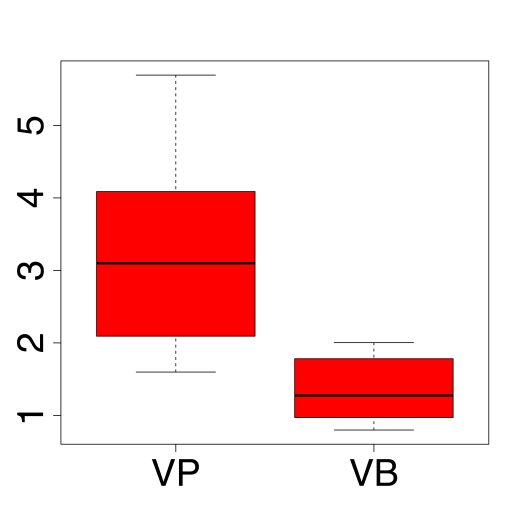

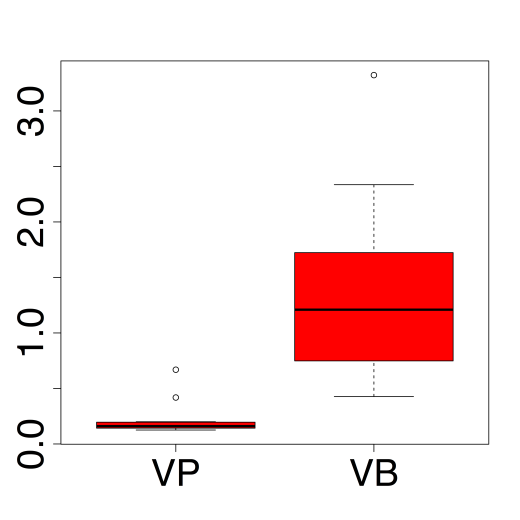

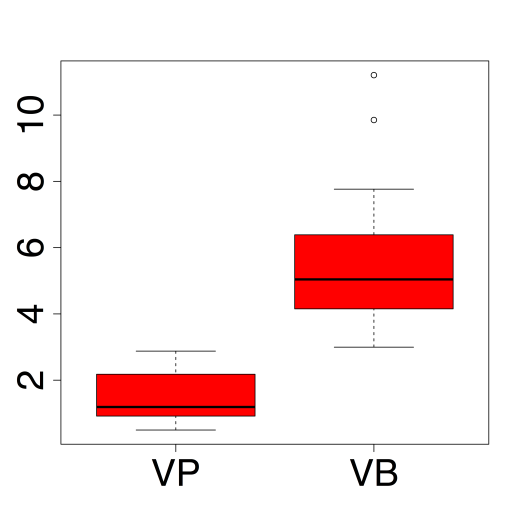


h

g

f

e


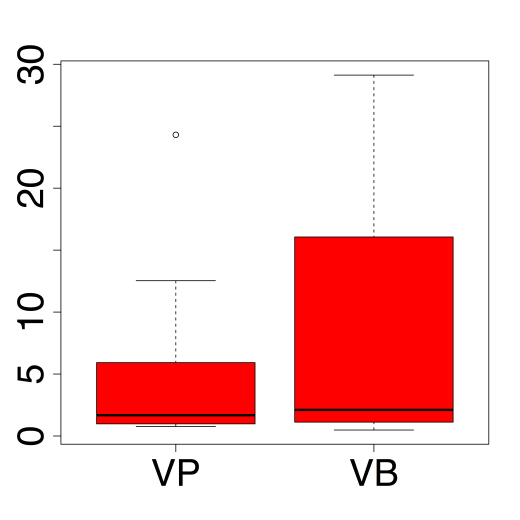

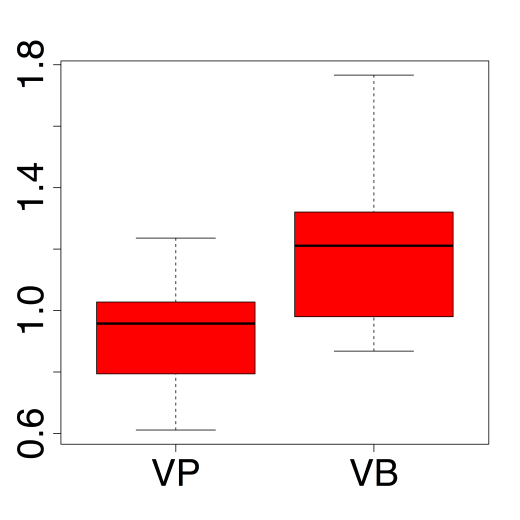

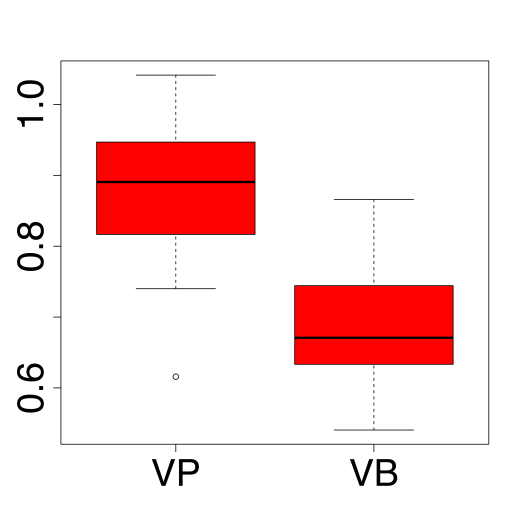

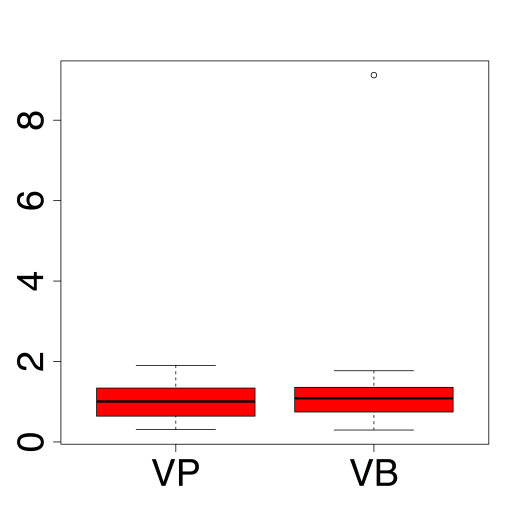


l

k

j

i


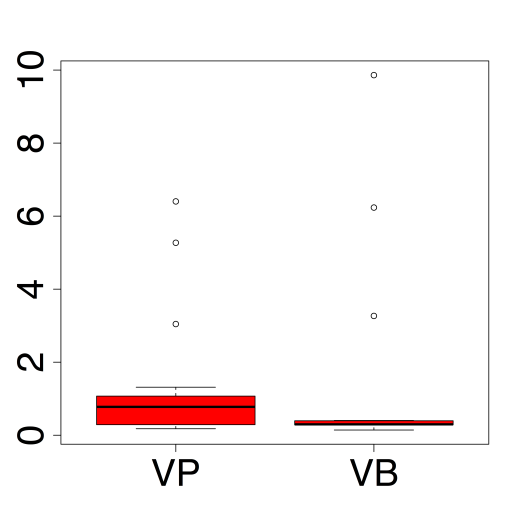

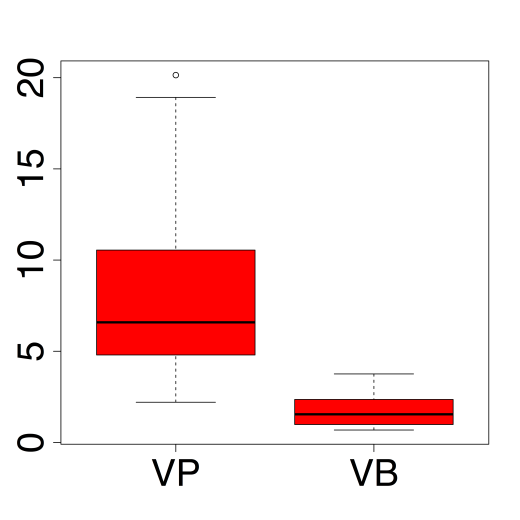

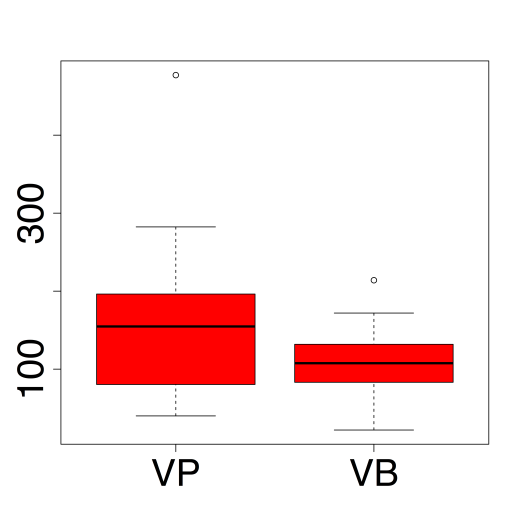

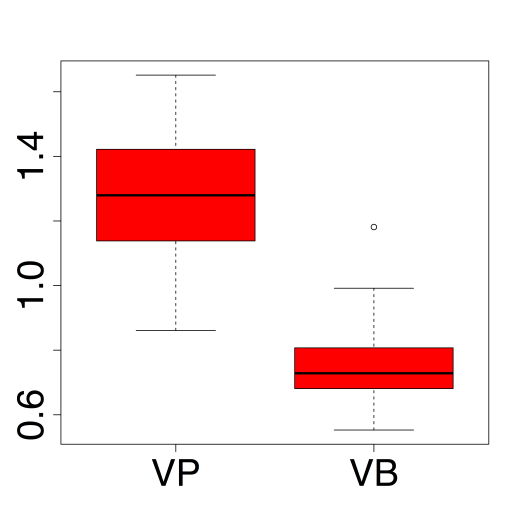


p

o

n

m


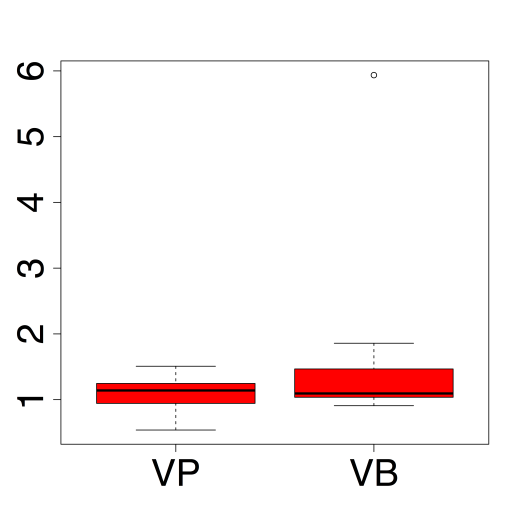

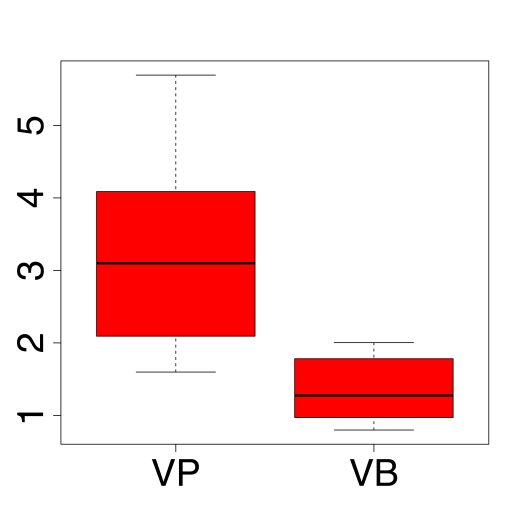

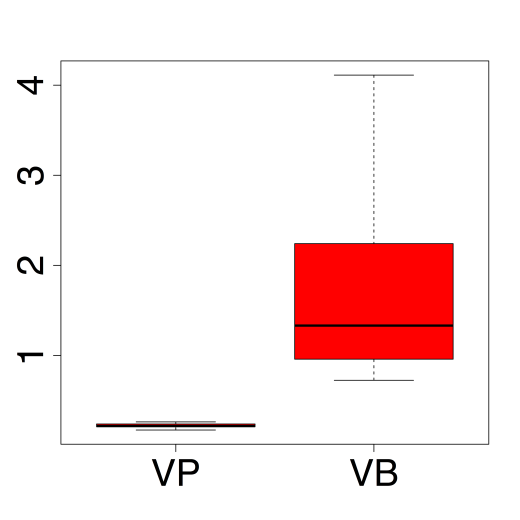

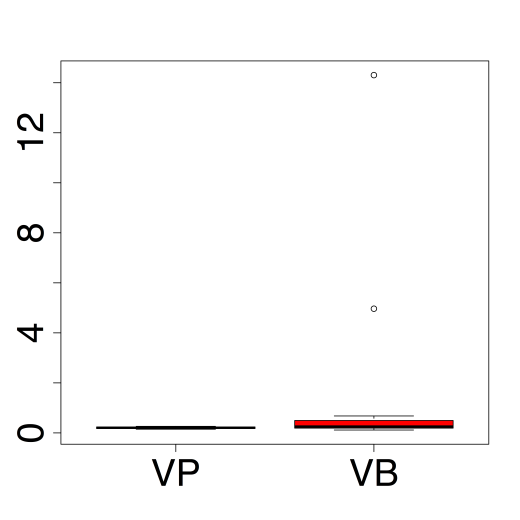


t

s

r

q


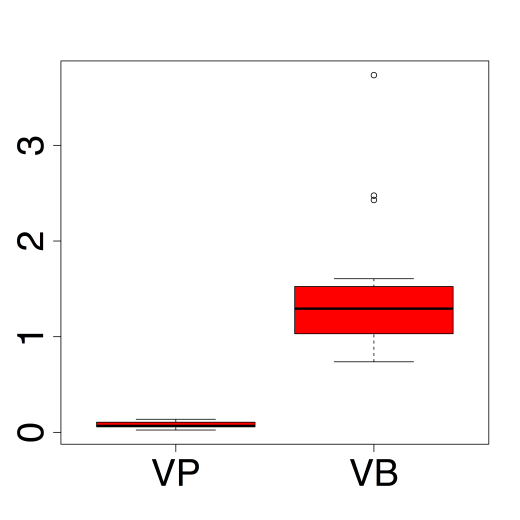

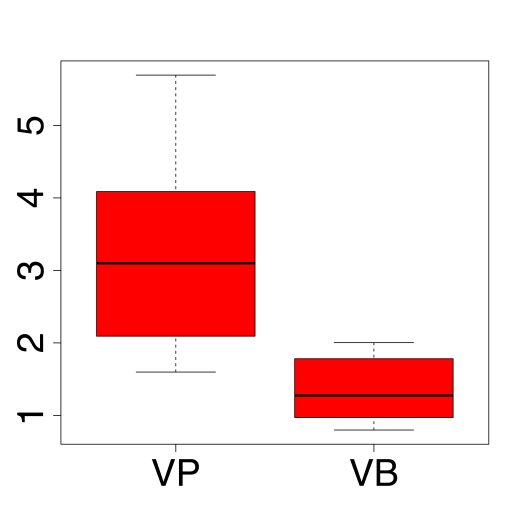

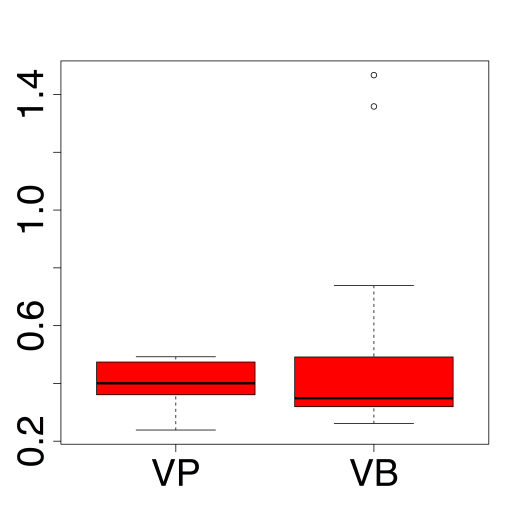

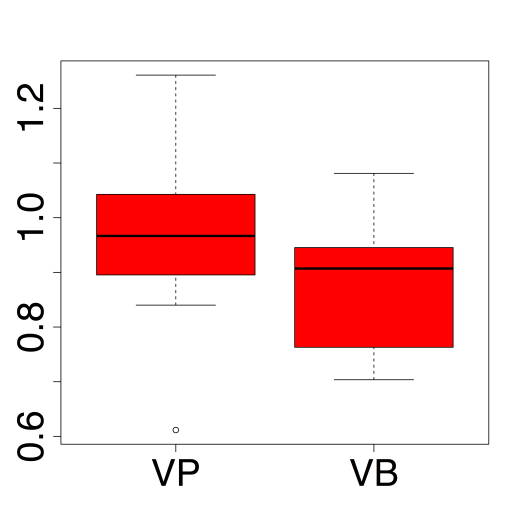


Additional file 1: Figure S1. A part of components change in the process and relate to the vanillin biosynthesis.

a. Erythrose-4-phosphate; b. Glucose; c. *p*-Hydroxybenzaldehyde; d. 3,4-Dihydroxybenzaldehyde; e. Protocatechuic acid; f. Cresol; g. 3-Dehydroshikimate; h. Vanillin; i. Vanillic acid; j. Vanillyl alcohol; k. Vanillylamine; l. Cinnamic acid; m. Phenylpyruvic acid, *p*-Coumaric acid; n. Cafferic acid; o. L-Phenylalanine; p. Tyrosine; q. Ferulic acid; r. Coniferyl alcohol; s. Glucovanillin; t. Capsaicin.
